# Turing Patterns Inside Cells

**DOI:** 10.1371/journal.pone.0001053

**Published:** 2007-10-17

**Authors:** Damián E. Strier, Silvina Ponce Dawson

**Affiliations:** 1 Service de Chimie Physique and Center for Nonlinear Phenomena and Complex Systems, Université Libre de Bruxelles, Brussels, Belgium; 2 Departamento de Física, Facultad de Ciencias Exactas y Naturales, Ciudad Universitaria, Pabellón I, University of Buenos Aires, Buenos Aires, Argentina; University of Nottingham, United Kingdom

## Abstract

Concentration gradients inside cells are involved in key processes such as cell division and morphogenesis. Here we show that a model of the enzymatic step catalized by phosphofructokinase (*PFK*), a step which is responsible for the appearance of homogeneous oscillations in the glycolytic pathway, displays Turing patterns with an intrinsic length-scale that is smaller than a typical cell size. All the parameter values are fully consistent with classic experiments on glycolytic oscillations and equal diffusion coefficients are assumed for *ATP* and *ADP*. We identify the enzyme concentration and the glycolytic flux as the possible regulators of the pattern. To the best of our knowledge, this is the first closed example of Turing pattern formation in a model of a vital step of the cell metabolism, with a built-in mechanism for changing the diffusion length of the reactants, and with parameter values that are compatible with experiments. Turing patterns inside cells could provide a check-point that combines mechanical and biochemical information to trigger events during the cell division process.

## Introduction

Concentration gradients inside the cytosol are a vital piece of the cell's machinery. They underlie morphogenesis [1], and are involved in cell migration [2], cell growth [3], cell division [4], and the mechanical responses that follow fertilization, among other functions [5]. Stationary concentration gradients can spontaneously emerge from a homogeneous background in the presence of instabilities. In 1952 Alan Turing proposed a hypothetical sequential route for cell differentiation, based on a reaction-diffusion process [6]. Starting from a spatially uniform state he proved that stable inhomogeneities in “morphogen” concentration could spontaneously emerge through a diffusion-driven symmetry-breaking instability. His highly idealized model stimulated a large amount of work which eventually led to the observation of *Turing patterns* in open chemical reactors [7], [8]. Nevertheless, the occurrence of Turing patterns has not been unequivocally proven for a biochemical reaction in which reaction rates, concentrations and diffusion coefficients are within realistic physiological values. We do so in this paper. More specifically, we show the existence of cell-sized Turing patterns in a model of glycolysis, using realistic parameter values and equal diffusion coefficients of *ATP* and *ADP*.

The general concept behind Turing patterns involves a combination of short-range activation and large-range inhibition [9]. Several reaction-diffusion models have been proposed in the literature to explain, among others, coat patterns in mammals [9], skin patterns in fish [10] or shell patterns in mollusks [11]. However, none of these models identifies the “morphogens” involved and some of the reaction schemes lack a biological motivation. This gives almost unrestricted freedom to choose kinetic parameters and diffusion ratios. Thus, the astonishing visual similarities often observed between the predicted and the real patterns might be unrelated to the actual biological phenomena.

One of the early attempts to look for an example of a symmetry breaking mechanism in biology was due to I. Prigogine *et al.* [12]. They analyzed a spatially extended version of the (adiabatically reduced) 2-variable Selkov model of glycolysis, derived in [13] (see also [14], [15]). Namely, they added diffusion terms to each of the two differential equations describing the time evolution of the concentrations of *ATP* and *ADP*. It followed from their study that Turing patterns could only exist provided that *ATP* and *ADP* diffused at sufficiently unequal rates. This condition seems difficult to be met *a priori*, given the similarities between *ATP* and *ADP*. More recently, Hasslacher *et al.* [16] presented numerical simulations of Turing pattern formation in a closely related model [17] that were obtained assuming that *ATP* diffused 25 *times faster* than *ADP*. The authors gave a qualitative justification for their choice of diffusivities making an analogy between the role of enzymes in the glycolytic pathway and that of the immobile color indicators used for visualization in open chemical reactors [7], [8] which effectively reduce the transport rate of one of the species involved in the reactions [18]. However, the argument that applied to the latter case cannot be translated to the case of the glycolytic pathway without a deeper analysis. Firstly, enzymes affect both the dynamics of *ATP* and *ADP*. Thus, the rescaling of diffusion coefficients may occur in such a way so as to prevent the formation of patterns. Second, when the 2-variable reaction-diffusion model is obtained by the adiabatic reduction of the larger set of equations in which the enzyme dynamics is explicitly considered [19], the elimination of the fast variables (the enzymes) introduces changes of the same order of magnitude in both the diffusion and the reaction terms [20]. Thus, both changes need to be considered simultaneously in order to analyze Turing pattern formation in this setting. Finally, this theoretical discussion remains meaningless unless reasonably sized patterns can be shown to exist for realistic values of the reaction rates and of the free diffusivities.

In this paper we overcome the drawbacks of these previous works by showing that the full 5-variable Selkov model describing the *PFK*-controlled steps of the glycolytic pathway supports Turing patterns for realistic reaction rates and equal diffusivities of *ATP* and *ADP*. We show that Turing structures of subcellular size (10 µm) may be found by increasing the glycolytic flux and the enzyme concentration while keeping fixed the set of kinetic constants that give good agreement with the classic experimental results on homogeneous oscillations in yeast extracts [21], [22], [23], [24], [14]. The emergence of these patterns can be traced back to the differential interactions of *ATP* and *ADP* with *PFK* and its complexes. We then conclude that the key enzymatic step responsible for glycolytic oscillations may also provide a robust mechanism for the formation of steady state inhomogeneities in the concentration of *ATP* and *ADP* at the cellular and supracellular level.

## Results

The 5-variable Selkov model reads [13]:
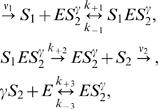
(1)where *E* is the free enzyme (*PFK*), which can form the complexes, *ES*_2_^γ^ and *S*_1_ *ES*_2_^γ^; the substrate, *S*_1_ (*ATP*) is supplied by an external source at the rate, ν_1_, and the product, *S*_2_^γ^ (*ADP*) is removed by a first order reaction at rate ν_2_[*S*_2_]. From scheme (1) it is clear that the enzyme is inactive unless it has γ product molecules bound, forming the complex *ES*_2_^γ^. As done in [13], we set γ = 2 all throughout this paper. It is this activating step of the allosteric enzyme which provides the mechanism responsible for the instabilities. We assume that *ATP* and *ADP* diffuse while the enzyme and its complexes are immobile, due to the larger mass. We also assume that, initially, all concentrations are spatially uniform. Using mass action kinetics, the reaction-diffusion equations describing this model can be written, in dimensionless form, as:
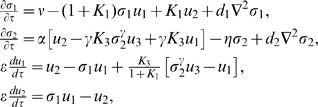
(2)where the dimensionless concentrations are:
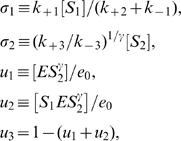
(3)with *e*_0_, the total concentration of enzyme which remains constant and uniform throughout the evolution. The other quantities are
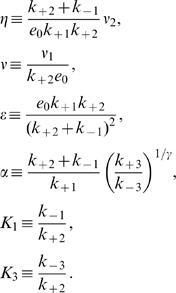
(4)The dimensionless space and time coordinates are obtained by dividing the dimensional ones by an arbitrary length-scale, *L*, and
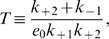
(5)respectively. Assuming that the diffusion coefficients of *ATP* (*D*_1_) and *ADP* (*D*_2_) are equal (*D≡D*_1_ = *D*_2_), we take *L*≡10*(DT)* ^1/2^. Using the diffusion coefficients *in vivo*, *D*∼150 µm^2^ s^−1^ [25], we obtain *L* = 122 µm (*T* s^−1^)^½^. while the dimensionless diffusion coefficients are *d*_1_ = *d*_2_ = 0.01. The relatively small amount of enzyme used in experiments implies that ε≪1. For this reason, a quasi-steady state approximation of Eqs. (2) was analyzed in [13] considering, furthermore, spatially uniform concentrations.

**Figure 1:**
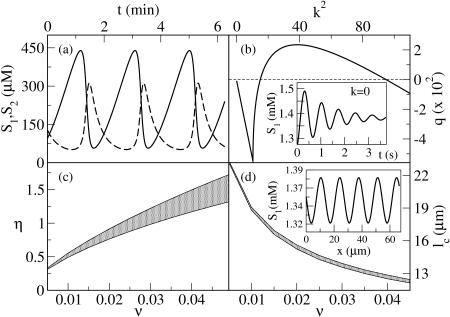
Behaviors predicted by the 5-variable Selkov model for different parameter values. (a) Glycolytic oscillations in *S*_1_ (solid curve) and *S*_2_ (dashed curve) for η = 0.15, ν = 0.00345, ε = 10^−6^, α = 15, *K*_1_ = 1500, *K*_3_ = 1. (b) Linear growth rate of the unstable modes as a function of the square of the wavenumber, *k* for η = 1.215, ν = 0.03, ε = 0.0003, α = 15, *K*_1_ = 1500, *K*_3_ = 1, and *d*_1_ = *d*_2_ = 0.01. Inset: Evolution of [*S*_1_] in the spatially homogeneous case for the same parameter values. (c) Turing space (shadowed domain) as a function of the (dimensionless) input and output rates of *ATP* (ν) and *ADP* (η), for the same parameter values as in (b). (d) Predicted value of the wave-length of the most unstable mode at each point in the Turing space of (c).

We now constrain the values of the various parameters of Eqs. (2) according to previous estimations and measurements [13], [15], [26], [21], [22], [23]. Under the experimental conditions of [22], in which experiments are done using yeast extracts, the values of ν_1_ and ν_2_ at which the oscillations start are ν_1_*∼5.8 µMs^−1^ and ν_2_*∼0.04 s^−1^ [15]. The enzyme is very diluted in these experiments, with *e*_0_* between 3 and 10 µM, the average concentrations of *ATP* and *ADP* are [*S*_1_*] = 630 µM and [*S*_2_*] = 150 µM, respectively, while the period of the oscillations, *T*, is between 3 and 5 min [15]. It has been shown unequivocally that the transition to oscillatory behavior in glycolysis is due to a *Hopf bifurcation* [24]. This means that the transition can be generically encountered by varying only one parameter. Equations (2) have a single homogeneous fixed point solution that depends on several parameters, which indeed undergoes a Hopf bifurcation. Thus, there are various ways by which this bifurcation can be reached. Among them, we choose the set of parameter values that are compatible with the observed frequency of oscillations and *ATP* and *ADP* concentrations. In particular, we find that at η = η* = 0.15, ν = ν*≈0.0041, ε = ε* = 10^−6^, α = 15, *K*_1_ = 1500, *K*_3_ = 1, a Hopf bifurcation occurs for the set of Eqs. (2) in the spatially homogeneous case. Using the definitions of these dimensionless quantities in terms of dimensional ones and the experimentally determined values, ν_1_*∼5.8 µMs^−1^ and ν_2_*∼0.04 s^−1^, we obtain that the total amount of enzyme at the Hopf bifurcation is e_0_ = e_0_*∼7.9 µM and that the various reaction rates satisfy
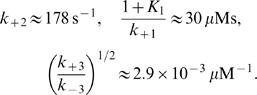
(6)These numbers imply that, at the Hopf bifurcation, the stationary homogeneous solution of Eqs. (2) occurs at [*S*_1_*]∼150 µM and [*S*_2_*]∼145 µM, and that the dimensional period of the oscillations is T*∼2.7 min, which agrees with the experimentally determined values. We show the oscillatory behavior of [*ATP*] and [*ADP*] close to this bifurcation in Fig. 1 (a).

Given these previous estimates, we explore the behavior of Eqs. (2), in the spatially extended case, varying the parameters ν, η, and ε and keeping the purely kinetic constants, α, *K*_1_ and *K*_3_ fixed at the previously mentioned values. We obtain the set of parameter values for which the homogeneous stationary solution of Eqs. (1) is unstable against spatially inhomogeneous perturbations (*i.e.*, the *Turing space*) by analyzing the dispersion relation of the linearized evolution equations. We show in Fig. 1(b) the (dimensionless) linear growth rate, *q*, of the unstable modes as a function of the square of the (dimensionless) wavenumber, *k*, for ν = 0.03, η = 1.215, ε  = 0.0003. There is a bounded band of unstable modes for *k*≠0, while for *k* = 0 (see the inset) the fixed point is stable. We show in Fig. 1(c) the Turing space on the (ν,η) plane for ε = 0.0003. As it may be observed, patterns may exist for larger values of ν_1_ as ν_2_ also gets larger, *i.e.*, as the glycolytic flux increases. A similar picture holds on the (ε,η) plane (data not shown): no pattern is possible at low enzyme concentration (if ε≤10^−4^). We show in Fig. 1(d) the wave-length of the most unstable mode, l_c_, when η and ν are varied in the shaded region of Fig. 1(c). We then conclude that the characteristic size gets smaller as the rate of product removal, ν_2_, becomes larger. We observe that, for the parameter values considered, the length-scale is always less than 23 µm, so that it can fit inside a typical cell. Using the definition of ε with the value of *K*_1_ = 1500 and the previous rate constant estimates (Eqs. (6)) obtained at the Hopf bifurcation, which we assume remain fixed, we conclude that *e*_0_≥800 µM for the Turing instability to occur. As we discuss later, the patterns arise due to the effective rescaling of the diffusion coefficients of *ADP* and *ATP* that the enzyme produces. This rescaling gets smeared out as e_0_ decreases, leading, in turn, to the disappearance of the patterns.

**Figure 2:**
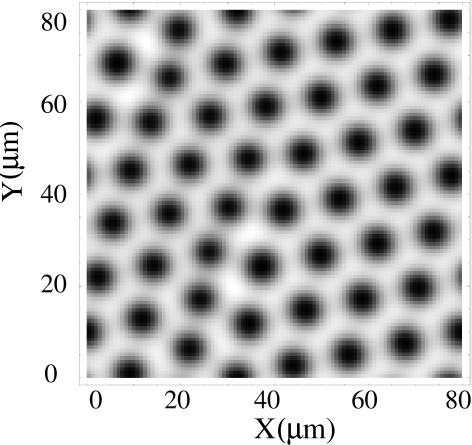
Turing pattern obtained with the 5-variable Selkov model in two space dimensions. Stationary pattern in [*ATP*] achieved after 10 min from an initial condition randomly distributed around the Turing-unstable fixed point. White corresponds to [*ATP*] = 2.47 mM and black to 1.1 mM. The simulation was done for η = 1.3, ν = 0.0175, ε = 0.0005, α = 15, *K*_1_ = 1500, *K*_3_ = 1, and *d*_1_ = *d*_2_ = 0.01.

We finally integrate numerically Eqs. (2) in a square domain of side *8L* = 84.5 µm with periodic boundary conditions, using a finite-difference scheme on a 150×150 square grid. Fig. 2 shows the resulting pattern in *S*_1_ after 10 min have passed since the initial situation, at which the concentrations are given by the homogeneous stationary solution ([*S*_1_] = 2.4 mM and [*S*_2_] = 71 µM), with 10% added noise. The typical size of the pattern agrees with the critical wavelength of the linear stability analysis, l_c_ ≈12 µm. The simulation shows that Eqs. (2) support stable Turing patterns with equal diffusion coefficients of *ATP* and *ADP*. The existence of these patterns can be understood in terms of the reduced set of equations obtained in the quasi-steady state approximation [19], [20], [27]. The *effective* diffusion coefficients of *ATP* and *ADP* that are obtained in this approximation are different due to the different interaction of *ATP* and *ADP* with the enzymes that belong to the pathway. In this scheme, *ATP* cannot bind to the immobile activated complex unless γ = 2 *ADP* molecules are already bound. Thus, the effective diffusivity of *ADP* –which plays the role of the activator of the step– is reduced by a larger amount than that of *ATP*.

## Discussion

### Conlusions

In this paper we have provided the first closed example of Turing pattern formation in a model of a vital piece of a cell's real biochemistry, with a built-in mechanism for the change of the morphogens diffusion length, and with parameter values that are compatible with experiments. Our results suggest that the pattern of enzyme regulation that gives rise to the glycolytic oscillations may also provide the basis for the formation of stationary spatial structures both at the cellular and supracellular level. The model we have used is highly idealized and cannot account for certain observations. However, we think that some of its basic dynamical features should be common to those of more sophisticated models [22], [28] and of the real system. In particular, it does reproduce the oscillations that are observed for dilute enzyme concentrations [22], [23], and has helped the finding of rotating spirals *in vitro* [29]. There are studies that show that the Turing and Hopf bifurcations are intimately related in systems with immobile species [30]. Thus, we expect all these models to share the existence of a Turing bifurcation at large enough (immobile) enzyme concentration and a Hopf bifurcation at lower ones. Our study has shown that the interactions involved in the *PFK* catalyzed step of the glycolytic pathway change the “effective” diffusion coefficients of *ATP* and *ADP* in the necessary direction for Turing pattern formation. These results could be tested in the type of open reactors that have especially been conceived for the investigation of spatio-temporal dynamics in glycolysis [31].

We have also found that the patterns can fit inside a typical cell and that the time it takes for the patterns to form is relatively short (of the order of minutes). The formation of Turing patterns in this biochemical pathway could then be related to organizing centers in eukaryotic cells, playing a role during cell division [4]. The fact that the Turing patterns have an intrinsic length-scale implies that there could be zero, one or several spots of high [*ATP*] inside the cell, depending on the relationship between the cell and the pattern sizes (see Fig. 2). In particular, there are two properties which imply that the number of high [*ATP*] spots could change during the cell division cycle. First, the pattern size decreases as the strength of the glycolitic flux increase. This flux must increase during interphase for biosynthesis and growth. Thus, the pattern size might become small enough to fit inside the cell only after the glycolytic flux has increased sufficiently. The change of the cell size also acts in the same direction, allowing more “room” for more high [*ATP*] spots to fit as the cell grows during interphase. In this way, the change in the number of high [*ATP*] spots as the cell grows could provide a check-point that combines mechanical information (cell size) and biochemistry to trigger the subsequent chain of events in the cell division process. If a key stage of the replicating machine of every eukaryotic cell strongly relies on a metabolic pathway, it would be unlikely that such pathway had evolved after the appearance of the first eukaryotic cell. The pathway should be old and highly conserved. The glycolytic pathway shares both properties.
